# Supplementary material for: Temporal dynamics of the neural representation of hue and luminance polarity
Source: Nat Commun. 2022 Feb 3;13:661. doi: 10.1038/s41467-022-28249-0 (PMC8814185; doi:10.1038/s41467-022-28249-0)
Supplement: Supplementary file 1 — Supplementary Information [file 41467_2022_28249_MOESM1_ESM.pdf]

## SUPPLEMENTARY TABLES and FIGURES

## Temporal dynamics of the neural representation of hue and luminance polarity

Katherine L. Hermann[1*^], Shridhar R. Singh[1*], Isabelle A. Rosenthal[1*#], Dimitrios Pantazis[2], Bevil R. Conway[1,3, †]

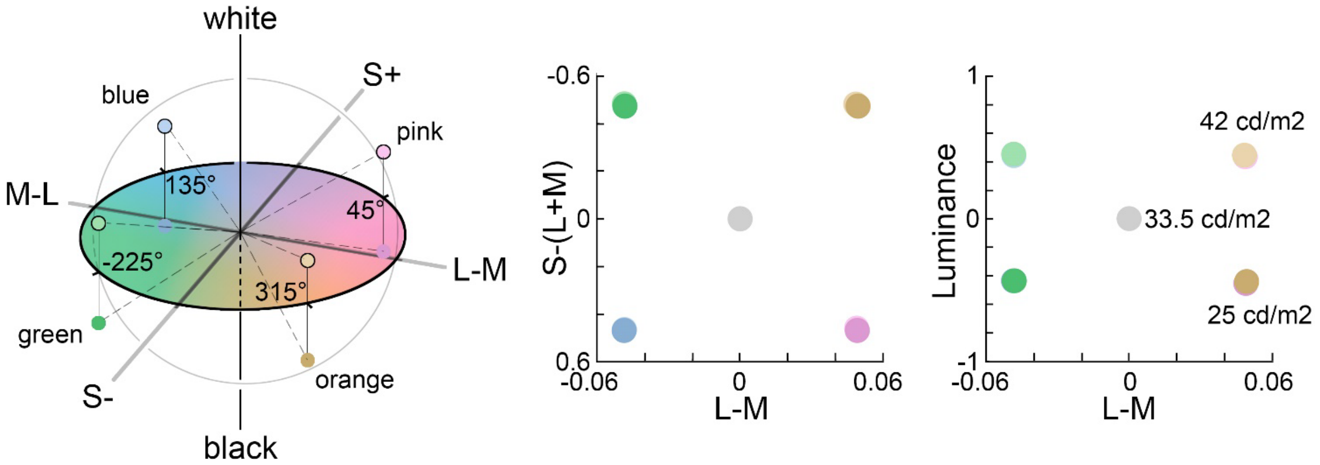


**Supplementary Figure 1.** Stimulus specification. Stimuli colors were set to the intermediate directions of the DKL color space, which is defined by the cardinal cone-opponent mechanisms.[1, 2] The left panel shows the eight colors at hue angles of 45°, 135°, 225°, and 315°, each hue at two luminance levels (elevations). The CIE xyY values for the stimuli are in SI Table 1. The DKL values were obtained using the function *lms2dkl* from Westland,[3] with the input LMS values computed in Supplementary Table 2. The right panels show the cone and luminance contrast of the stimuli, relative to the gray adapting background, with the luminance values in cd/m$^2$ indicated.

| | CIE x | CIE y | CIE Y' | Y |
|---|---|---|---|---|
| Light pink | 0.305229324 | 0.293448787 | 0.628205196 | 41.8950 |
| Dark pink | 0.304095045 | 0.264040315 | 0.370334398 | 24.6976 |
| Light blue | 0.272115611 | 0.307026806 | 0.630601697 | 42.0548 |
| Dark blue | 0.25265696 | 0.284531124 | 0.375699736 | 25.0554 |
| Light green | 0.310023683 | 0.426308239 | 0.63358717 | 42.2539 |
| Dark green | 0.312628272 | 0.502281154 | 0.375716373 | 25.0565 |
| Light orange | 0.352384707 | 0.400239965 | 0.631544192 | 42.1177 |
| Dark orange | 0.392438648 | 0.444792187 | 0.374272604 | 24.9602 |
| Adapting gray | 0.306634746 | 0.346610046 | 0.502314308 | 33.4993 |

**Supplementary Table 1.** The xyY values for the stimuli. The values in the third column (Y') yield luminance (Y), when multiplied by the maximum gun output (66.69 cd/2).

| | L | M | S |
|---|---|---|---|
| Light pink | 27.6333 | 14.2617 | 0.9216 |
| Dark pink | 16.5006 | 8.1970 | 0.6497 |
| Light blue | 26.7305 | 15.3243 | 0.9271 |
| Dark blue | 15.7224 | 9.3330 | 0.6554 |
| Light green | 26.8587 | 15.3952 | 0.4203 |
| Dark green | 15.7255 | 9.3310 | 0.1485 |
| Light orange | 27.7748 | 14.3429 | 0.4187 |
| Dark orange | 16.6733 | 8.2869 | 0.1469 |
| Adapting gray | 21.6919 | 11.8074 | 0.5389 |

**Supplementary Table 2.** The LMS coordinates of the stimuli, computed using the MATLAB function *xy2MB* from Westland.[3]

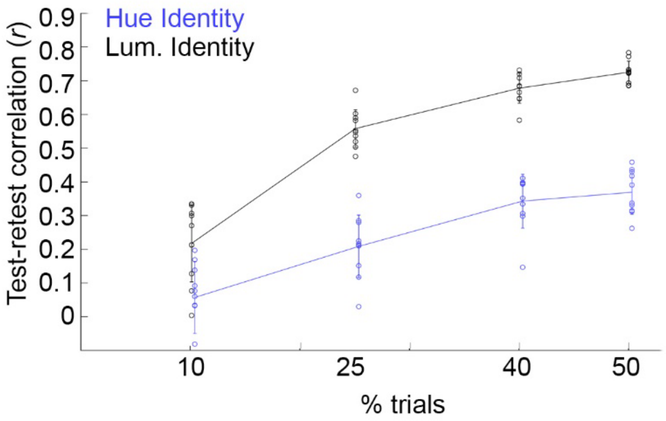


**Supplementary Figure 2.** Data reliability analysis for the pilot experiment (n=4 participants). The graph shows the correlation between pairs of independent data sets, as a function of the amount of data in the data set, for decoding hue identity and luminance-polarity identity. For each problem, pairs of classifiers were trained and tested on independent samples containing 10%, 25%, 40%, and 50% of the data, and the correlation between the classifiers' performance at each time point was calculated (i.e., the analysis shows the extent to which the shape of the classification curve is similar for independent data sets of different sizes). This procedure was repeated ten times to obtain the average test-retest correlation (center of error bars), and error bars, which represent the standard deviation across the ten replicates. This analysis provides a measure of data reliability.[4] The y-axis shows the correlation coefficient comparing the decoding magnitude at each point in the decoding time course between the pairs of data sets. The graph shows: (1) that the data are reliable (the test-retest estimates are above chance); (2) that the experiments have sufficient power to extract close to as much signal as is possible given the experimental conditions (the test-retest curves come close to plateau when extrapolated to 100% of the data); and (3) that to decode color, one needs relatively many trials. As in the main experiment, the stimuli were spirals, participants viewed each color 500 times, and each stimulus appeared for 100 ms followed by a 1 s ISI. Other details of experimental paradigm were the same as for the main experiment, with the following exceptions: the data were collected in 4 participants (3 female; separate people from those who participated in the main experiments); and the stimuli only included four colors of either a luminance increment or luminance decrement. The two hues were DKL angles 150 and 300, which are intermediate colors corresponding roughly to blue and yellow; the luminance of the stimuli were: background gray, 41 cd/$m^2$; positive luminance-contrast stimuli, 48-50 cd/$m^2$; and negative luminance-contrast stimuli, 30-32 cd/$m^2$).

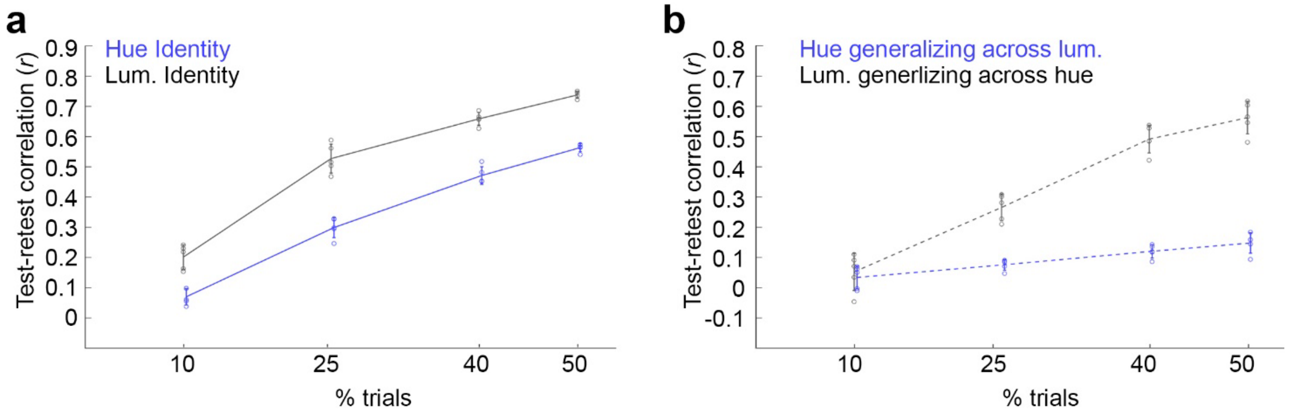


**Supplementary Figure 3.** Data reliability analysis for main experiments. **a)** Results for the identity problems. For each identity problem, pairs of classifiers were trained and tested on independent samples containing 10%, 25%, 40%, and 50% of the data, and the correlation between the classifiers' performance at each time point was calculated (the analysis shows the extent to which the shape of the classification curve is similar for independent data sets of different sizes). This procedure was repeated five times to obtain the average test-retest correlation (center of error bars) and error bars, which represent the standard deviation across the five replicates. **b)** Results for the generalization problems. Again, the correlation procedure was repeated five times to obtain the average test-retest correlation and standard deviation error bars. The test-retest curves in panel (a) and (b) differ somewhat from those in Supplementary Figure 2, which shows the same analysis for the pilot experiment. The data for the main experiments were obtained in n=18 participants using 8 colors, whereas the data for the pilot experiment were obtained in four participants using 4 colors.

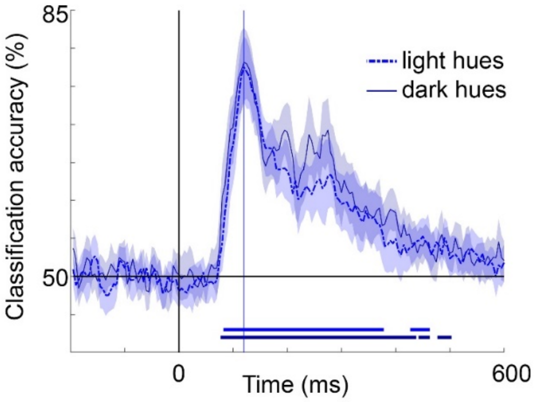


**Supplementary Figure 4.** The average classification accuracy (center of the shading) for the 6 sets of hue identity problems between stimuli of the same luminance polarity (six problems among light hues; six problems among dark hues). Shading represents the 95% confidence intervals on the accuracy, which were obtained by bootstrapping 18 subjects 1000 times. The time to peak was not significantly different for identity problems among luminance-contrast increments (122 ms [115, 130]) compared to identity problems among luminance-contrast decrements (123 ms [115, 130]); and the magnitude of peak decoding accuracy for these two sets of problems also were not significantly different (for light stimuli: 77% [73, 82]; for dark stimuli: 78% [74, 83]). The horizontal sequence of data points above the x-axis shows time bins at which decoding was significantly above chance, FDR corrected, for a minimum of four consecutive time bins.

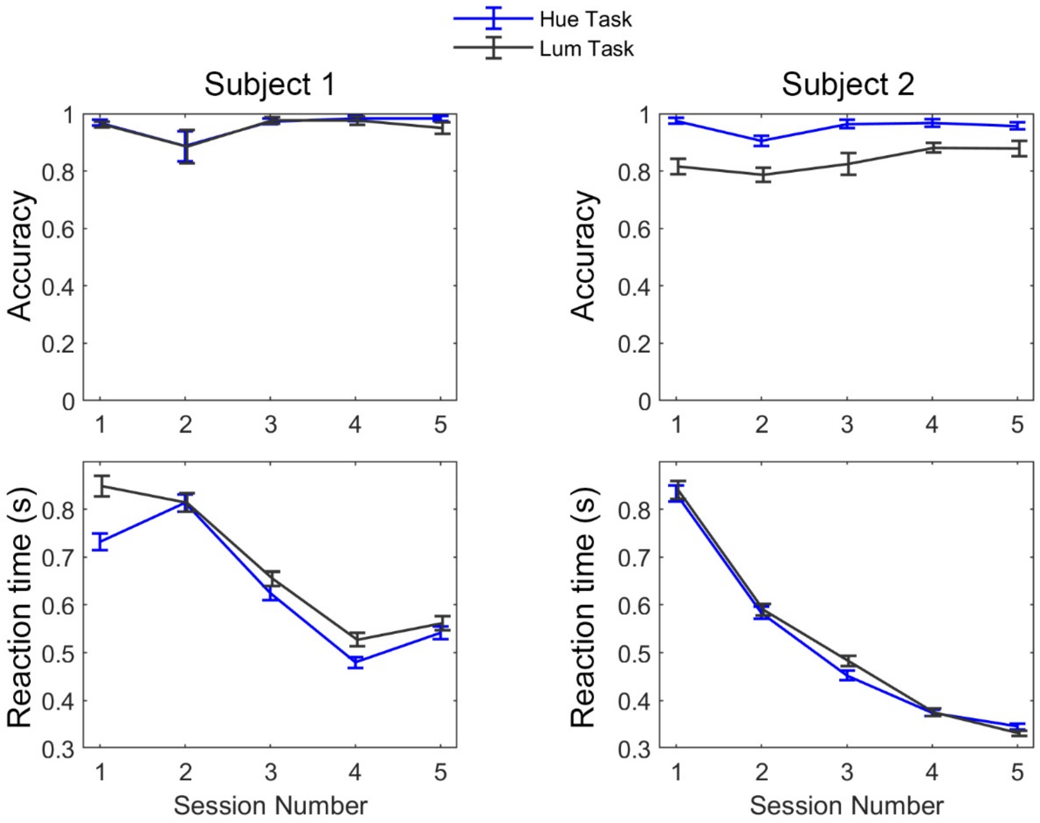


**Supplementary Figure 5.** Behavioral performance for the two subjects from the control experiment in which MEG data were collected while participants performed either the 1-back luminance-polarity matching task (black traces) or the 1-back hue matching task (blue traces; see Figure 5 for the MEG decoding results of this control experiment). Note the slightly longer reaction times for the luminance task (Subject 1) and the slightly lower accuracy for the luminance task (Subject 2), which reflects the general difficulty people face in making heterochromatic luminance-contrast matches. For subject 1, error bars and averages were obtained from n=249, 253, 248, 248, 251 trials for each session respectively. For subject 2, the number of trials per session were n=248, 254, 248, 248, 252. Error bars represent the standard error across trials.

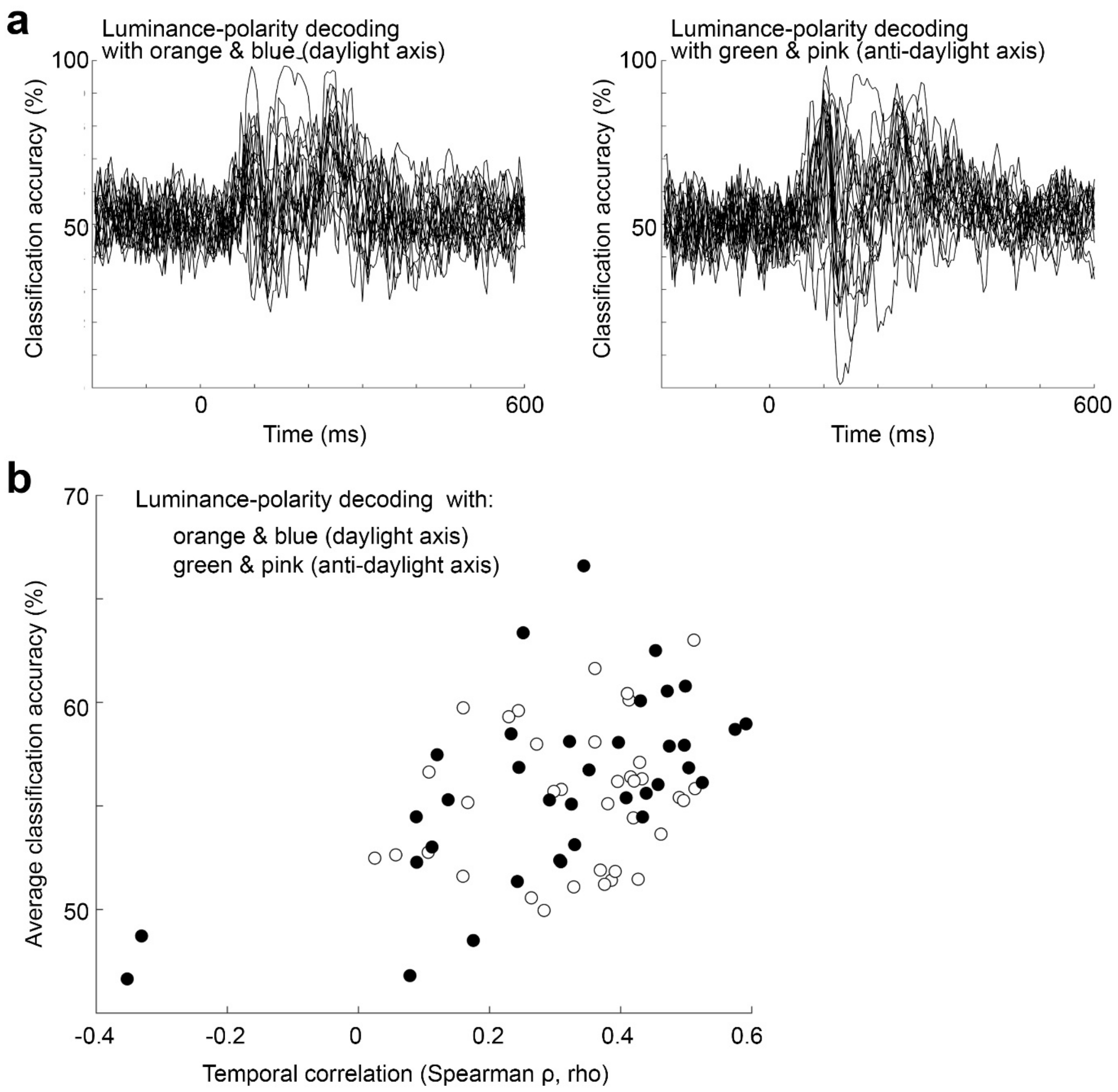


**Supplementary Figure 6.** Luminance-polarity decoding was more variable across participants for problems using orange and blue versus problems using green and pink. **a)** Individual luminance-polarity decoding curves for problems involving orange and blue (left panel) and pink and green (right panel) across the 18 participants (36 problems illustrated in each panel, two per participant involving orange and blue; and two per participant involving pink and green). The decoding curves using pink and green were more correlated across time, as quantified in panel (b). **b)** The average classification accuracy (averaged over the entire decoding time course from 0 to 600 ms) for each problem, versus the temporal correlation of the problem. The temporal correlation for each problem was computed by measuring the correlation between it and a problem drawn at random from the remaining 71 problems, repeated 1000x, and averaged. Decoding luminance polarity was more variable for data obtained using orange and blue compared to data obtained with green and pink (the black dots are shifted to the right; one-way MANOVA, $p=0.0007$).

Supplementary References


1. MacLeod DI, Boynton RM. Chromaticity diagram showing cone excitation by stimuli of equal luminance. *J Opt Soc Am* **69**, 1183-1186 (1979).

2. Derrington AM, Krauskopf J, Lennie P. Chromatic mechanisms in lateral geniculate nucleus of macaque. *Journal of Physiology* **357**, 241-265 (1984).

3. Westland S, Ripamonti C, Cheung V. Computational colour science using matlab (2nd Edition). *John Wiley and Sons Ltd, The Atrium, Southern Gate, Chichester, West Sussex, England*, (2012).

4. Norman-Haignere SV, Kanwisher N, McDermott JH, Conway BR. Divergence in the functional organization of human and macaque auditory cortex revealed by fMRI responses to harmonic tones. *Nature Neuroscience* **22**, 1057-+ (2019).
