# Supplementary material for: Temporal dynamics of the neural representation of hue and luminance polarity
Source: Nat Commun. 2022 Feb 3;13:661. doi: 10.1038/s41467-022-28249-0 (PMC8814185; doi:10.1038/s41467-022-28249-0)
Supplement: Supplementary file 3 — Reporting Summary [file 41467_2022_28249_MOESM3_ESM.pdf]

nature research

Corresponding author(s): Bevil Conway

Last updated by author(s): Dec. 20, 2021

Reporting Summary

Nature Research wishes to improve the reproducibility of the work that we publish. This form provides structure for consistency and transparency in reporting. For further information on Nature Research policies, see our Editorial Policies and the Editorial Policy Checklist.

Statistics

For all statistical analyses, confirm that the following items are present in the figure legend, table legend, main text, or Methods section.

n/a	Confirmed	
☐	☒	The exact sample size (*n*) for each experimental group/condition, given as a discrete number and unit of measurement
☐	☒	A statement on whether measurements were taken from distinct samples or whether the same sample was measured repeatedly
☐	☒	The statistical test(s) used AND whether they are one- or two-sided<br>*Only common tests should be described solely by name; describe more complex techniques in the Methods section.*
☒	☐	A description of all covariates tested
☐	☒	A description of any assumptions or corrections, such as tests of normality and adjustment for multiple comparisons
☐	☒	A full description of the statistical parameters including central tendency (e.g. means) or other basic estimates (e.g. regression coefficient) AND variation (e.g. standard deviation) or associated estimates of uncertainty (e.g. confidence intervals)
☐	☒	For null hypothesis testing, the test statistic (e.g. *F*, *t*, *r*) with confidence intervals, effect sizes, degrees of freedom and *P* value noted<br>*Give P values as exact values whenever suitable.*
☒	☐	For Bayesian analysis, information on the choice of priors and Markov chain Monte Carlo settings
☒	☐	For hierarchical and complex designs, identification of the appropriate level for tests and full reporting of outcomes
☐	☒	Estimates of effect sizes (e.g. Cohen's *d*, Pearson's *r*), indicating how they were calculated

Our web collection on statistics for biologists contains articles on many of the points above.

Software and code

Policy information about availability of computer code

Data collection	Stimuli were presented using MATLAB (2016) code utilizing Psychtoolbox3. The experiment was run using Matlab 2016.
Data analysis	MEG data were preprocessed using Brainstorm3 and Elekta Neuromag's MaxFilter version 2.1 software. Decoding was accomplished using custom code written in MATLAB as well as the Neural Decoding Toolbox. MRI data were analyzed using Freesurfer 5.3 and custom MATLAB (2016) scripts. The custom MATLAB scripts will be available on NEICOMMONS at the following URL: https://neicommons.nei.nih.gov/#/decodingColorWithMeg

For manuscripts utilizing custom algorithms or software that are central to the research but not yet described in published literature, software must be made available to editors and reviewers. We strongly encourage code deposition in a community repository (e.g. GitHub). See the Nature Research guidelines for submitting code & software for further information.

Data

Policy information about availability of data

All manuscripts must include a data availability statement. This statement should provide the following information, where applicable:

- Accession codes, unique identifiers, or web links for publicly available datasets
- A list of figures that have associated raw data
- A description of any restrictions on data availability

Source data are provided with this paper. The MEGco data and analysis in this study have been deposited in the OpenNeuro data base and the NEICOMMONS databases under accession codes https://openneuro.org/datasets/ds003352/versions/1.0.0, https://neicommons.nei.nih.gov/#/MEGco

Field-specific reporting

Please select the one below that is the best fit for your research. If you are not sure, read the appropriate sections before making your selection.

- [ ] Life sciences
- [x] Behavioural & social sciences
- [ ] Ecological, evolutionary & environmental sciences

For a reference copy of the document with all sections, see nature.com/documents/nr-reporting-summary-flat.pdf

Behavioural & social sciences study design

All studies must disclose on these points even when the disclosure is negative.

Study description	MEG and MRI data were collected from human subjects. All subjects provided informed consent and were compensated financially ($30/hour). The data were quantitative. Subjects viewed colored spirals while performing a behavioral task. MEG data was collected while subjects viewed the spirals and performed the task. Before MEG data collection, subjects performed a different behavioral task where they were instructed to name the color of spirals shown to them.
Research sample	There were 18 subjects, ages 19-37, 11 of whom were female. *A* The sample is representative of healthy young adults with normal (or corrected-normal) vision. The subjects were recruited via email sent through the MIT participants mailing list. All subjects spoke English as a first language, had no neurological or psychiatric conditions, had normal or corrected-to-normal vision, and had normal color vision. The sample was chosen because this study is concerned with the dynamics of normal color processing in humans, and the sample is representative of people with normal color vision. The raw dataset collected from the experiment is the MEG signal acquired while the subjects viewed colored spirals. It is available at https://doi.org/10.18112/openneuro.ds003352.v1.0.0
Sampling strategy	The sampling procedure was determined on the basis of a prior study (Rosenthal et al, Current Biology, 2021), which itself was based on convenience. No sample-size calculation was performed, but the number of participants is consistent with sample sizes in other MEG papers, and we performed a power analysis to evaluate test-retest reliability (see SI Material). The goal was to find 16-20 participants based on the number of participants involved in previously published studies on vision utilizing MEG (https://doi.org/10.1016/j.neuroimage.2017.07.023).
Data collection	MEG data were collected on an Elekta Triux system. Eye tracking data were collected using an an Eyelink 1000 Plus eye tracker. MRI data were collected using a Siemens 3T MAGNETOM Prisma fit scanner. The researcher was present in the control room while the subjects were scanned. The researchers were not blind to the study hypothesis, but the subjects were.
Timing	Pilot data were acquired in 2014 and 2015; the Main experiments were conducted from 2015-2017 (there was no sustained gap in collection of data; data sessions were scheduled throughout the years listed).
Data exclusions	The data collected from one subject was excluded because it contained too many artifacts, likely due to building construction occurring outside the MEG laboratory at the time.
Non-participation	19 subjects dropped out either due to scheduling difficulties or lack of interest.
Randomization	Participants were not allocated into experimental groups.

Reporting for specific materials, systems and methods

We require information from authors about some types of materials, experimental systems and methods used in many studies. Here, indicate whether each material, system or method listed is relevant to your study. If you are not sure if a list item applies to your research, read the appropriate section before selecting a response.

Materials & experimental systems

n/a	Involved in the study	
x		Antibodies
x		Eukaryotic cell lines
x		Palaeontology and archaeology
x		Animals and other organisms
	x	Human research participants
x		Clinical data
x		Dual use research of concern

Methods

n/a	Involved in the study	
x		ChIP-seq
x		Flow cytometry
	x	MRI-based neuroimaging

Human research participants

Policy information about studies involving human research participants

Population characteristics	See above
Recruitment	Participants were recruited via a list of research participants and by email. Self-selection bias should not impact our results because the study concerned normal visual response, and participants were screened for normal color vision.
Ethics oversight	Wellesley College Institutional Review Board, the Massachusetts Institute of Technology Committee on the Use of Humans as Experimental Subjects, and the National Institutes of Health Intramural Institute Clinical Research Review Committee

Note that full information on the approval of the study protocol must also be provided in the manuscript.

Magnetic resonance imaging

Experimental design

Design type	Task based. Block design
Design specifications	Participants completed 8 runs of the task, each containing 25 blocks of 18 seconds.
Behavioral performance measures	Subjects pressed a button.

Acquisition

Imaging type(s)	Functional
Field strength	3T
Sequence & imaging parameters	Pulse sequence: T2 weighted image; Imaging type: EPI; FOV: 192 mm; Matrix size: 96x96 mm; Slice thickness: 2 mm; Orientation: parallel to temporal lobe; TE: 30 ms; TR: 2 ms; Flip angle: 90 degrees
Area of acquisition	The area of acquisition spanned the ventral surface of the temporal lobe.

Diffusion MRI ☐ Used ☒ Not used

Preprocessing

Preprocessing software	Data were preprocessed using Freesurfer 5.3. The data were field corrected, motion corrected using rigid-body transformations to the middle of each run, intensity normalized, and spatially smoothed using a 3 mm FWHM isotropic Gaussian kernel
Normalization	All subjects' anatomical images were aligned to a template using the combined volumetric and surface based (CVS) registration algorithm in Freesurfer. This algorithm includes a non-linear registration step.
Normalization template	The subjects' anatomical images were aligned to Freesurfer's cvs_avg35 template in Talairach space.
Noise and artifact removal	Motion correction was accomplished using Afni's 3dvolreg function, which uses a rigid body, 6 parameter transformation. EPI's were dewarped with an echo spacing of 0.69 ms and an echo time difference of 2.46 ms.
Volume censoring	There was no volume censoring.

Statistical modeling & inference

Model type and settings	The analysis was mass-univariate with a fixed effects model.
Effect(s) tested	Stimulus conditions were videos of faces, scenes, objects, and scrambled objects. Additional conditions were chromatic and achromatic gratings. Contrasts were created for faces versus objects, scenes versus objects, objects versus scrambled objects, and color versus black and white. This was a categorical design.

Specify type of analysis: ☐ Whole brain ☐ ROI-based ☒ Both

Anatomical location(s)	Retinotopic visual areas were obtained by registering each person's brain to a standard anatomical atlas in Freesurfer, and generating visual area ROIs using automated parceling.
Statistic type for inference (See Eklund et al. 2016)	Voxel-wise comparison
Correction	Correction for multiple comparisons was not necessary for this experiment.

Models & analysis

n/a	Involved in the study
☒	☐ Functional and/or effective connectivity
☒	☐ Graph analysis
☒	☐ Multivariate modeling or predictive analysis
